# Development and psychometric characteristics of the SCI-QOL Bladder Management Difficulties and Bowel Management Difficulties item banks and short forms and the SCI-QOL Bladder Complications scale

**DOI:** 10.1179/2045772315Y.0000000030

**Published:** 2015-05

**Authors:** David S. Tulsky, Pamela A. Kisala, Denise G. Tate, Ann M. Spungen, Steven C. Kirshblum

**Affiliations:** 1 Department of Physical Therapy, University of Delaware, Newark, DE, USA; 2 Kessler Foundation Research Center, West Orange, NJ, USA; 3 Department of Physical Medicine and Rehabilitation, University of Michigan Medical School, University of Michigan, Ann Arbor, MI, USA; 4 VA RR&D Center of Excellence for the Medical Consequences of Spinal Cord Injury, James J. Peters VA Medical Center, Bronx, NY, USA; 5 Departments of Medicine and Rehabilitation Medicine, Icahn School of Medicine at Mount Sinai, NY, NY, USA; 6 Kessler Institute for Rehabilitation, West Orange, NJ, USA; 7 Department of Physical Medicine and Rehabilitation, Rutgers - New Jersey Medical School, Newark, NJ, USA

**Keywords:** Spinal cord injuries, Urinary bladder, Neurogenic, Neurogenic bowel, Patient reported outcomes assessment, Quality of life, Psychometrics

## Abstract

**Objective:**

To describe the development and psychometric properties of the Spinal Cord Injury – Quality of Life (SCI-QOL) Bladder Management Difficulties and Bowel Management Difficulties item banks and Bladder Complications scale.

**Design:**

Using a mixed-methods design, a pool of items assessing bladder and bowel-related concerns were developed using focus groups with individuals with spinal cord injury (SCI) and SCI clinicians, cognitive interviews, and item response theory (IRT) analytic approaches, including tests of model fit and differential item functioning.

**Setting:**

Thirty-eight bladder items and 52 bowel items were tested at the University of Michigan, Kessler Foundation Research Center, the Rehabilitation Institute of Chicago, the University of Washington, Craig Hospital, and the James J. Peters VA Medical Center, Bronx, NY.

**Participants:**

Seven hundred fifty-seven adults with traumatic SCI.

**Results:**

The final item banks demonstrated unidimensionality (Bladder Management Difficulties CFI = 0.965; RMSEA = 0.093; Bowel Management Difficulties CFI = 0.955; RMSEA = 0.078) and acceptable fit to a graded response IRT model. The final calibrated Bladder Management Difficulties bank includes 15 items, and the final Bowel Management Difficulties item bank consists of 26 items. Additionally, 5 items related to urinary tract infections (UTI) did not fit with the larger Bladder Management Difficulties item bank but performed relatively well independently (CFI = 0.992, RMSEA = 0.050) and were thus retained as a separate scale.

**Conclusion:**

The SCI-QOL Bladder Management Difficulties and Bowel Management Difficulties item banks are psychometrically robust and are available as computer adaptive tests or short forms. The SCI-QOL Bladder Complications scale is a brief, fixed-length outcomes instrument for individuals with a UTI.

## Introduction

Individuals with spinal cord injury (SCI) are living longer and surviving at 70–92% of average life expectancy.^1,2^ As living with a disability becomes a life-long process for many persons with SCI, different sets of problems are likely to occur at different stages in life. Given the complexity of the issues involved in managing SCI across the years, the need for lifelong follow up is important in order to better understand and promote physical, psychological, and social well-being after SCI.^3^ These factors provide a clear rationale for the current interest in quality of life (QOL) of individuals with SCI. QOL can be clearly influenced by the need for neurogenic bladder and bowel management, its disruptions to lifestyle routines, and the potential occurrence of related complications.

### Effects of neurogenic bladder and bowel on quality of life

The vast majority of those with SCI and neurologic impairment also have associated bladder and bowel dysfunction making this aspect of their care relevant to providers, researchers, individuals with SCI and their families and caregivers.^4^ In contrast, very few studies have focused on the impact of neurogenic bladder and bowel on QOL or behavioral factors associated with bladder and bowel dysfunction. Qualitative findings from a study conducted by Tate *et al.*^5^ include many testimonials of the negative effects of bladder and bowel dysfunction on QOL. These testimonials refer to difficulties in finding accessible bathrooms for performing intermittent catheterization or bowel management, feelings of embarrassment, consequences to personal relationships, marriage, sexuality, and intimacy, and obstacles to maintaining regular employment.

Although the scientific literature is limited on the impact of neurogenic bladder and bowel on people's lives, the few clinical studies on this topic confirm statements made by SCI participants from the study above. For women with tetraplegia, bladder management is particularly challenging as noted by Walsh *et al.*^6^ Anecdotal clinical evidence also suggests that persons with SCI often have difficulty maintaining social relationships due to fear of accidents.^7,8^ The importance of coping mechanisms and lifestyle changes that can affect QOL for those with neurogenic bowel is highlighted in a 2004 article by Rockwood.^9^ The author suggests that psychological mechanisms such as coping play an important role in QOL following fecal incontinence, as it contributes to the overall measure of incontinence severity. Specific psychosocial modules of assessment are needed to provide a more complete evaluation of the impact of bowel dysfunction on QOL.

### Bladder and bowel dysfunction and related medical complications

Bladder dysfunction refers mostly to voiding problems and abnormalities in bladder function.^10^ The signs and symptoms of bladder dysfunction are variable and can change.^11^ Although population-specific QOL instruments have been developed for SCI, urological issues are not always well reflected in the scores due to the broad nature of questions. The role that patients take in their own health care is key when assessing bladder management issues after SCI.^12^ The need is great for a patient reported outcome (PRO) measure which assesses urological symptoms and consequences from a subjective perspective.^13,14^ Measures like the Qualiveen,^15^ which offers specific questions for SCI and has strong psychometric properties, tend to focus on feelings related to bladder dysfunction.^16^

Neurogenic bowel dysfunction is usually characterized by lower gastrointestinal (GI) symptoms such as loss of voluntary control over bowel movements, difficulty with evacuation, and incontinence. These symptoms represent major physical and psychological problems for individuals with SCI, as changes in bowel motility and sphincter control coupled with impaired mobility and hand dexterity make bowel management a major life-limiting problem. Thus, it is not surprising that improving bowel function alone is rated as one of the highest priorities among individuals with SCI.^17,18^

Medical complications from neurogenic bladder and bowel can be severe and include presence of constipation, incontinence, urinary tract infections (UTIs), fecal impaction, pain, pressure ulcers secondary to leakage, renal failure, and bladder and kidney stones.^19^ Severe UTIs are one of the most frequent reasons for re-hospitalizations after SCI.^20–22^

It is well established that neurogenic bowel dysfunction significantly impacts the QOL and health of persons with SCI.^23–25^ In addition to fecal incontinence, constipation is a particularly common and bothersome GI complaint in persons with SCI.^26–28^ Patients with SCI and chronic constipation report a variety of symptoms including straining, a sensation of incomplete evacuation, reduced stool frequency, a sensation of anorectal blockage and the need for ‘manual maneuvers.’^29^ GI issues also have been reported to worsen with time.^23,24,30^ Moreover, abdominal or rectal pain occurring from distension and constipation has been known to trigger autonomic dysreflexia,^24,30^ and up to 25% of individuals with SCI require at least one re-hospitalization due to GI tract issues.^30^ Despite the high frequency of complications, there are very few controlled studies related to bowel management. The lack of specific measures that assess PROs in relation to bowel dysfunction is one of the reasons for the paucity of such studies. A few measures are currently available including the Patient Assessment of Constipation Symptom questionnaire (PAC-SYM) and the Patient Assessment of Constipation Quality of Life (PAC-QOL), more specifically assessing QOL in relation to symptoms.^31,32^ Neurogenic bowel dysfunction (NBD) can be assessed by a score as proposed by Krogh *et al.* (2006) which includes items on fecal incontinence, constipation, obstructed defecation and impact and QOL.^33^ The NBD score was found to be significantly associated with impact on QOL. Finally, the Revised Faecal Incontinence Scale (RFIS) scale may be used to assess fecal incontinence and monitor patient outcomes, though only one item is devoted to the effects of incontinence on QOL.^34^

Urological complications have been reported to account for much of the SCI associated morbidity and as much as 15% of the SCI associated mortality.^35^ These complications often lead to social problems, which may in turn decrease QOL. Restoration and maintenance of the patient's QOL should remain among the main targets of the treatment.^36^

Evidence is mixed regarding methods of bladder management being associated with complications.^37–39^ A systematic review of urological follow-up after SCI was conducted to investigate various methods of screening for bladder complications with the ultimate goal of creating practice guidelines.^40^ Based on this review, only routine renal ultrasound could be recommended as part of screening. While more sophisticated urodynamic evaluations seem important to consider, evidence is lacking.

A 2011 study using SCI Model Systems data by Pelletier-Cameron *et al.* found that the use of an indwelling catheter was associated with more medical complications, including pressure ulcers and re-hospitalizations.^41^ Pannek and Kullik suggest that a treatment regimen leading to favorable urodynamic outcome and continence was associated with better QOL.^42^ When considering medical factors, older age seems to be suggestive of a higher number of complications. The more chronic the lesion and the older the patient becomes, the less attention the patient receives in the community and the more he/she is likely to develop complications.^43^

Unlike bladder management, there are no clearly distinct bowel management methods. Instead, adequate bowel management strategies consist of a combination of dietary, pharmacological and manual methods of producing a controlled bowel evacuation. Recommendations such as a high-fiber diet and adequate hydration, prescribed treatments of oral laxatives and stool softeners or rectal suppositories, and manual methods, such as digital rectal stimulation for manual evacuation or flushing enemas, are used depending on each person's functional ability and health needs. This lack of uniformity in bowel management programs and the lack of tools to measure change in bowel function is perhaps a methodological reason for difficulties in conducting research in this area. Hence, the need for patient reported measures with respect to bowel management.

### Behaviors and environmental support factors associated with complications

Many with SCI consider their bowel management to be a very arduous process since it may necessitate help from a caregiver, can take as long as one hour or more to achieve, and can be accompanied by autonomic dysreflexia, bleeding hemorrhoids and rectal pain.^26,44^ Furthermore, despite best efforts there often remains the increased risk of an unwanted or unplanned bowel evacuation. Bowel management often requires a combination of time, expense, and quality of caregiver support.^45^ Clinical experience suggests that for those with SCI who have less upper extremity physical function, the availability of and quality of caregivers are critical factors in predicting ability to maintain health and prevent complications. Health adherence behaviors and environmental support based on relationships such as caregiving and patient provider relations are important to consider when examining modifiable factors that may influence bladder and bowel complications after SCI.

Important issues affecting bowel and bladder management behaviors include depression, anxiety and fear of incontinence, which can significantly limit an individual's ability to engage in activities outside the home.^17^ Other concerns include increased stress and withdrawal, lack of independence, and difficulties with sexuality and intimacy.^17,46^ From an environmental support perspective, the implementation of a bladder and/or bowel management program poses additional challenges to the individual with SCI, as this often requires assistance from family, friends or caregivers; special equipment; use of accessible environments; medications; and dietary changes. Such programs can be time consuming and difficult to perform, some requiring exposure to unpleasant and embarrassing events, thus at times generating further stress within already overtaxed relationships and/or role reversals among partners or other family members.^47^ Research has shown that spouses who provide care for persons with SCI report feeling significantly more physical and emotional stress, burnout, fatigue, anger and resentment in comparison to non-caregiving spouses.^48,49^ Sexuality and intimacy are often affected by these bowel and bladder problems.^4^ The experience of increased stress on the part of both caregivers and persons with SCI may in turn make it more difficult to do what is necessary to prevent complications by interfering with healthy behaviors critical to successful bladder and bowel management, such as following a proper diet and adhering to prescribed treatments.^7^

In their review of bowel management in SCI, Steins and colleagues make an important recommendation relevant for our proposed research (and one that equally applies to bladder management): ‘It is crucial to remember that the patient must take a leadership role in building a bowel program that incorporates a life-compatible bowel care schedule. Our job is to educate him or her about altered physiology after SCI and to empower him or her to construct a bowel care regimen that he/she can live with’ (p. S86).^44^ In order to achieve ‘life-compatible’ bowel care, a better understanding of the environment, behavior and personal characteristics is needed.

## Methods

The Institutional Review Boards at each of the 6 sites reviewed and approved this study. The first study activity was to develop and refine pools of items related to bladder and bowel issues that affect health-related quality of life (HRQOL). Next, bladder and bowel items were administered to a large, stratified sample of individuals with traumatic SCI. Items were administered in interview format and a computerized data collection platform was used to capture data in real time. Psychometric analyses included confirmatory factor analyses (CFA), graded response model^50^ item response theory (IRT) analyses, and tests of differential item functioning (DIF).^51^ Each of these steps is described in detail in the introductory^52^ and methods^53^ papers in this issue, and is also outlined briefly in the section below.

### Development of bladder and bowel item pools

To develop the bladder and bowel item pools, candidate items from initial pilot work were identified. These items were obtained via individual, semi-structured interviews and focus groups with individuals with SCI and SCI clinicians (see Tulsky *et al.*^54^ for a full description). The interview data informed the development of a set of 26 preliminary items related to bladder and/or bowel related concerns. Relevant concepts or phrases were then drawn from the focus group transcripts and converted into 135 additional ‘new’ items. For example, from quotes like ‘*I get a UTI* *…* *every couple of months*’ and ‘*I had [UTIs] for 3 months ongoing*’ we drafted the items, ‘I had a urinary tract infection’ and ‘I had a urinary tract infection that would not go away.’

One-hundred and sixty-one preliminary items underwent Expert Item Review (EIR),^55^ a method in which several project co-investigators reviewed each item for issues such as relevance and clarity and made suggestions for item revisions and deletions. Based on EIR feedback, 52 bladder items and 72 bowel items were retained. These items then underwent an additional phase of review and modification in which the items were arranged on a hierarchy, from items indicating the lowest degree of bladder or bowel-related problems to the highest degree. Team members removed redundant items where there was oversaturation in the middle range of the item hierarchies, and suggested new items to fill gaps in content coverage. During this phase of review, 5 bladder items were removed.

These refined sets of SCI-QOL bladder and bowel items were evaluated by individuals with SCI during structured, debriefing cognitive interviews (CI).^56^ CI participants were first asked to respond to each item, then describe the process they used to state their answer, and report whether they perceived anything to be confusing, unclear, or derogatory, or whether they thought any items should be reworded. Three bladder items and 10 bowel items were modified, and 9 bladder items and 3 bowel items were deleted based on CI feedback. Notably, 6 bowel items and 2 bladder items were added based on topics suggested by CI participants. For example, participants felt that the item pool lacked content related to the inconvenience associated with bowel accidents (e.g. *‘My clothing was soiled due to a bowel accident’*; *‘I spent a lot of time taking care of a bowel accident’*). After this phase, the final 38 bladder and 58 bowel items were reviewed for translatability (for method, please see Eremenco *et al.*),^57^ and reading level (using the Lexile framework).^58^ Slight modifications were made to 1 bladder item and 3 bowel items after the translatability and cultural review. For example, the item *‘I was troubled by constipation’* was changed to *‘I was bothered by constipation,’* since translation of the word ‘*troubled*’ could be ambiguous in this context. All items were written at the 5th grade reading level.

### Calibration study participants and data collection procedures

As a part of a large-scale multi-site item calibration study (sites included the Kessler Foundation, University of Michigan, Rehabilitation Institute of Chicago, University of Washington, Craig Hospital and the James J. Peters VA Medical Center), we administered the 38 bladder items and 58 bowel items along with other item pools reflecting other physical-medical subdomains to a sample of people with SCI.

The calibration sample included 757 individuals with traumatic SCI. Inclusion criteria were 18 years of age and older, ability to read and understand English, and had a documented SCI of traumatic etiology. The sample was stratified by level of injury (paraplegia, tetraplegia), completeness of injury (neurologically complete, incomplete), and time since injury (<1 year, 1–3 years, and >3 years) to ensure heterogeneity of the final sample. Each participant's diagnosis was confirmed by medical record review; neurologic level was documented by their most recent American Spinal Injury Association Impairment Scale (AIS) rating.^59^ All items were presented in a structured interview to participants in person or over the phone. Additional detail on the field testing methodology for this study is presented in Tulsky *et al.*^53^

### Data analyses

IRT is a family of mathematical models that are used to scale a pool of test items along a single underlying metric. In this way, the score on any subset of items in this calibrated ‘item bank’ is directly comparable to either the full bank score or to the score on any other subset of items. IRT scaling is a prerequisite to the development of computer adaptive tests (CAT). Analysis of the field testing data involved assessment of construct unidimensionality, use of a graded-response IRT model to calibrate items, and examination of local item dependence (LID) and DIF. We used CFA to determine if our items conformed to a unidimensional model. The following indicators of model fit were used: Comparative Fit Index (CFI) >0.90, Root Mean Square Error of Approximation (RMSEA) <0.08, good support of the model; CFI > 0.95, RMSEA < 0.06, excellent support of the model. Analyses were performed iteratively to reduce the item pools and obtain the best-fitting item parameters that would best allow estimation of a participant's standing on the traits related to bladder and bowel, respectively. We examined LID to ensure that residual correlations between items did not exceed |0.20| (i.e. to ensure that items were measuring unique aspects of the construct and were not simply restatements of one another). DIF analyses were conducted to ensure that the final items were free from measurement bias—that is, that the probability of a participant receiving a certain score was based only on their level of the underlying trait (e.g. Bladder Management Difficulties) and was not affected by characteristics such as sex or level of injury. Specifically, DIF analyses flag items that perform differently (when matched on the underlying trait) among different subgroups of individuals in an unanticipated or unexplained way.

With each successive analytic iteration, we identified poorly fitting items by examining item fit to a graded response IRT model, DIF, LID (i.e. removal of items with residual correlations >|0.20|), and significant loadings on the single factor (i.e. removal of items with values <0.30). With each iteration we removed problematic items from the pools and repeated the analytic steps. Once an acceptable solution was reached with CFA statistics that supported a unidimensional model, and all items showing misfit to the model or DIF were removed, the final IRT parameters were utilized to develop CAT algorithms for the final item banks. The CATs were programmed on the Assessment Center^SM^ ^60^ website (http://www.assessmentcenter.net) and were administered directly from there. The item parameters were also used to select items for fixed-length short forms, which can either be administered directly through the Assessment Center website or downloaded as a PDF from the Assessment Center^SM^ website. Additional detail on the methodology and analysis plan may be found in Tulsky *et al.* within this special issue.^53^

### Test-retest reliability

As part of a larger study, test-retest reliability was assessed in a second sample of community-dwelling adults with traumatic SCI who had been injured at least 4 months prior, and discharged from inpatient rehabilitation at least 4 weeks prior to the first assessment. Participants completed all measures again at 1–2 weeks post baseline.

## Results

### Participant characteristics

Bladder Management Difficulties and Bowel Management Difficulties items and other item pools related to physical-medical health were administered to a sample of 757 individuals with SCI. Demographic and injury characteristics are summarized in Table 1. Additional detail on the SCI-QOL calibration sample, as well as on the sample used to compute test-retest reliability, may be found in Tulsky *et al.* (this issue).^53^

**Table 1:** SCI-QOL participant characteristics

Variable	Physical/Medical domain sample (*n* = 757)	Bladder complications subsample (*n* = 297)
Age (years)	42.9 ± 15.5	40.6 ± 15.2
Sex		
Male	79.1%	77.8%
Female	20.9%	22.2%
Ethnicity		
Hispanic	10.6%	12.8%
Non-Hispanic	87.8%	85.9%
Not Reported	1.6%	1.4%
Race		
Caucasian	71.1%	68.1%
Black or African-American	17.2%	16.9%
Asian	1.5%	2.4%
American Indian/Alaska Native or Native Hawaiian/Pacific Islander	0.9%	1.3%
More than one race	1.5%	0.7%
Other	6.8%	9.2%
Not Reported	1.1%	1.3%
Time since injury (years)	6.7 ± 9.9	7.6 ± 11.6
<1 year post injury	28.9%	27.6%
1–3 years post injury	27.6%	23.9%
>3 years post injury	43.5%	48.5%
Diagnosis		
Paraplegia complete	23.9%	31.1%
Paraplegia incomplete	18.5%	13.2%
Tetraplegia complete	23.1%	28.9%
Tetraplegia incomplete	34.4%	26.8%
Education level		
High school or less	38.4%	39.4%
Some college	33.5%	35.4%
Bachelor's degree or higher	28.1%	25.3%
Injury etiology		
Motor vehicle accident	32.4%	37.7%
Fall	22.3%	18.9%
Gunshot wound/violence	11.8%	13.5%
Diving	6.6%	5.4%
Other sports	7.4%	8.1%
Medical/Surgical accident	3.7%	2.7%
Motorcycle accident	2.6%	2.7%
Other or not reported	6.2%	10.5%
Method(s) of mobility (not mutually exclusive)		
Manual wheelchair	54.4%	55.9%
Power wheelchair	44.1%	49.8%
Ambulation	32.7%	13.1%

### Data analysis—bladder management difficulties

#### Preliminary analysis and item removal

Data analysis was initiated on a pool of 38 general ‘Bladder’ items related to both bladder management and bladder complications. Following the first round of preliminary analyses and CFA, 10 items were removed for the following reasons (some items were removed for multiple reasons): low item-total correlations (7 items), bimodal distribution (5 items), sparse (<5 respondents) categories (2 items), misfit (significant χ^2^ test, 1 item), LID (1 item). Analyses were re-run and an additional two items were removed due to very low item-total correlations (i.e. <0.3 and <0.2, respectively). After these preliminary iterations, fit statistics had not yet reached acceptable levels (CFI = 0.893, RMSEA = 0.089) so the item bank content was re-reviewed and the remaining items were ‘binned’ into 2 discrete sets of item subdomains. One set related to issues surrounding bladder management and the other to bladder-related medical complications such as urinary tract infections (UTIs). The investigative team binned a 19-item set related to bladder management difficulties and re-ran the analyses with just those items, then binned the additional 8 items (including one item that had previously been deleted due to LID) into a second subset of items that were related to bladder complications. The analysis for the 8 items related to bladder complications is reported in its own section later in the manuscript. For the bladder management difficulties set of items, 4 additional items were removed at this stage as follows: 2 items removed for poor/emotional wording *(‘I felt inadequate because of my bladder program’* and *‘I was disappointed I depended on others for help with my bladder program’*) and 2 items that didn't fit well with the others psychometrically (low item-total correlations). The following results are based on the final 15-item set. For the 15 items, internal consistency was α = 0.91 and item/total correlations ranged from 0.38 to 0.78. In terms of test-retest reliability, Pearson's *r* was 0.77 (*n* = 245, P < 0.01) and the intraclass correlation coefficient (ICC) (2,1) was 0.76 (*n* = 245; 95% CI = 0.70 to 0.81). All of the items had more than 50% of the sample selecting category of ‘1’ (*Never* or *Not at all*). No items had sparse data (i.e. <5 responses) in any category. Two items had a category inversion with the average raw score for which persons selecting category ‘5’ (Always/Very much) were lower than the average for person selecting category ‘4’ (Often/Quite a bit). No additional items were removed.

#### Dimensionality

Using CFA, a unidimensional model was confirmed (CFI = 0.965; RMSEA = 0.093). *R*^2^ values for 11 of the items were greater than 0.40 and 4 items were less than 0.40. In terms of local dependence, no item pairs were identified (i.e. residual correlations > |0.20|). Eigenvalue ratio (first to second) was 8.5.

#### IRT parameter, estimation, and model fit

Slopes (discrimination parameters) ranged from 1.05 to 4.21; thresholds (difficulty parameters) ranged from 0.10 to 3.23 (see Table 2).

**Table 2:** Bladder Management Difficulties items and IRT parameters

Item ID	Response set*	Item stem	Slope	Threshold 1	Threshold 2	Threshold 3	Threshold 4
**rToiletBL_1**	**A**	**Bladder management interfered with my sleep**	**1.16719**	**0.49669**	**1.32225**	**2.18143**	**2.98756**
**rToiletBL_5**	**A**	**I worried that I would have a bladder accident**	**2.76165**	**0.17128**	**0.82065**	**1.43408**	**1.83494**
**rToiletBL_16**	**A**	**I worried about performing my bladder program**	**1.61663**	**1.13981**	**1.85039**	**2.63301**	**3.22778**
**rToiletBL_23**	**A**	**I was sad / depressed because of problems with bladder functioning**	**2.04375**	**0.80860**	**1.43685**	**2.09764**	**2.59699**
**rToiletBL_30**	**A**	**Bladder accidents limited my independence**	**3.14633**	**0.89336**	**1.33440**	**1.84038**	**2.33832**
**rToiletBL_31**	**A**	**I was frustrated by bladder accidents**	**4.20586**	**0.55064**	**1.04089**	**1.49926**	**1.87459**
rToiletBL_32	A	I was bothered by urine leakage	2.86357	0.34123	0.92878	1.56539	2.14690
rToiletBL_39	B	I spent a lot of time taking care of my bladder program	1.36346	0.10172	0.79277	1.46572	2.04802
rToiletBL_42	B	I restricted my fluid intake in order to manage my bladder	1.04545	0.40994	0.92706	1.98277	2.97543
**rToiletBL_72**	**B**	**Bladder accidents have disrupted my daily activities**	**3.85401**	**0.66154**	**1.06174**	**1.82561**	**2.21694**
rToiletBL_73	B	I had to stop what I was doing because of a bladder accident	3.58787	0.58616	1.05381	1.83373	2.20367
**rToiletBL_76**	**B**	**I had bladder accidents**	**3.79297**	**0.35862**	**0.95416**	**1.67111**	**1.94110**
rToiletBL_80	C	I felt my bladder management was under control	1.27867	0.50109	1.57824	2.33976	2.54941
rToiletBL_Com11	B	I worried that I would have a bladder accident in public	2.49816	0.47227	1.04258	1.79473	2.27231
**rToiletBL_Com26**	**A**	**I worried about performing my bladder program in a public restroom**	**1.65331**	**1.17300**	**1.70896**	**2.39132**	**3.19058**

*Context for all items was: ‘Lately’. Response set A was: 1 = Not at all/2 = A little bit/3 = Somewhat/4 = Quite a bit/5 = Very much. Response set B was: 1 = Never/2 = Rarely/3 = Sometimes/4 = Often/5 = Always. C was: 1 = Always/2 = Often/3 = Sometimes/4 = Rarely/5 = Never.

**Bold Font** indicates the items selected for the short form.

All SCI-QOL Items Copyright © 2015 David Tulsky and Kessler Foundation. All Rights Reserved. Scales should be accessed and used through the corresponding author or http://www.assessmentcenter.net. Do not modify items without permission from the copyright holder.

**Figure 1:**
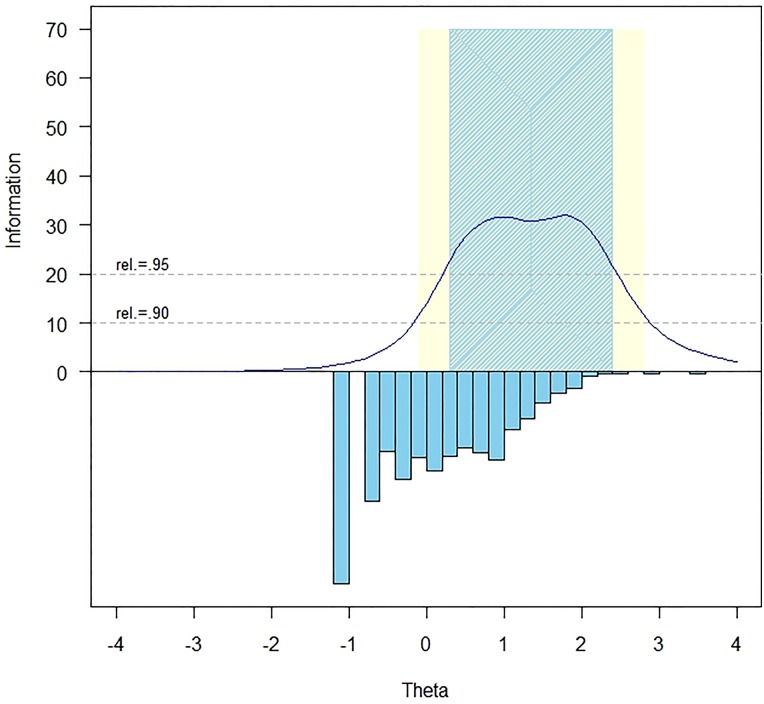
Bladder Management Difficulties item bank information and precision.

The measurement precision in the theta range between 0.3 and 2.4 is roughly equivalent to a classical reliability of 0.95 or better (Fig. 1).

The IRTFIT^61^ macro program was used to examine S-X^2^ model fit statistics. All but one item (rToiletBL_80, ‘I felt my bladder management was under control’, P < 0.01) had adequate or better model fit statistics (P > 0.05), with marginal reliability equal to 0.84.

#### Differential item functioning

The *lordif* ^62^ program was used to examine DIF for six categories: age (≤49 *n* = 494 vs. ≥50 *n* = 251), sex (male *n* = 599 vs. female *n* = 158), education (some college and lower *n* = 543 vs. college degree and above *n* = 212), diagnosis (tetraplegia *n* = 416 vs. paraplegia *n* = 307), injury severity (incomplete *n* = 383 vs. complete *n* = 340), and time post injury (<1 year *n* = 218 vs. >1 year *n* = 539). Items were flagged for possible DIF when the probability associated with the χ^2^ test was <0.01 and the effect size measures (McFadden's pseudo *R*^2^) >0.02, which is a small but non-negligible effect. Overall, 4 items were flagged for DIF in at least one category based on the χ^2^ test; however, when the effect size measures were examined, the DIF was negligible and all 19 items were retained in the final, calibrated item bank.

### Data analysis—bowel management difficulties

#### Preliminary analysis and item removal

Analyses began with the full pool of 58 items related to neurogenic bowel and its management. Following the first round of preliminary and CFA analyses, 16 items were removed for the following reasons (reasons for removal are not mutually exclusive): low item-total correlation (5 items), poor or ambiguous wording (5 items), bimodal distribution (4 items), LID (4 items), sparse category (<5 responses, 2 items), low slope (i.e. inability to discriminate between individuals at different levels of the underlying trait, 2 items), redundant with a better-performing item (2 items). Analyses were then re-run on the pool of 36 remaining items, and an additional 5 items were removed due to the following: LID (2 items), misfit (χ^2^ P < 0.05, 1 item), low item-total correlation (2 items). Analyses were repeated on the pool of 31 items, and an additional 4 items were removed (χ^2^ P < 0.05, 3 items; LID, 2 items; sparse categories, 1 item). Following analysis of this pool of 27 items, one final item was removed due to LID with two other items.

Following the above analyses, a final set of 26 items were retained. Internal consistency was α = 0.95 and item/total correlations ranged from 0.32 to 0.79. In terms of test-retest reliability, Pearson's *r* was 0.74 (*n* = 245; P < 0.01) and the ICC (2,1) was 0.74 (*n* = 245; 95% CI = 0.68 to 0.79). All of the items had more than 50% of the sample selecting category of ‘1’ (Never/Not at all). No items had sparse data (i.e. <5 responses) in any category. Two items had a category inversion with the average raw score for persons selecting category ‘5’ (Always/Very much) were lower than the average for person selecting category ‘4’ (Often/Quite a bit). No additional items were removed.

#### Dimensionality

Using CFA, a unidimensasional model was observed (CFI = 0.955; RMSEA = 0.078). *R*^2^ values for 22 of the items were greater than 0.40 and 4 items were less than 0.40. Eigenvalue ratio (first to second) was 9.8. In terms of local dependence, 1 item pair was identified with a residual correlation >|0.20| (rToiletBO_44, *‘Bowel care interfered with my sleep’* with rToiletBO_67, *‘My bowel program took 3 to 6 hours’*; *r* = 0.226). Since the items appear to be measuring 2 separate constructs, the research team decided to retain both items.

#### IRT parameter estimation and model fit

Slopes ranged from 0.92 to 4.90; thresholds ranged from –0.01 to 3.68 (see Table 3).

**Table 3:** Bowel Management Difficulties items and IRT parameters

Item ID	Response Set*	Item stem	Item response theory calibration statistics				
			Slope	Threshold 1	Threshold 2	Threshold 3	Threshold 4
rToiletBO_3	A	I worried about the odor associated with bowel accidents	2.31810	0.33615	0.81416	1.39188	1.75706
**rToiletBO_4**	**A**	**Bowel accidents limited my independence**	**3.15488**	**0.48635**	**1.02217**	**1.46920**	**1.87993**
**rToiletBO_7**	**A**	**A bowel accident has affected my self-esteem**	**2.85321**	**0.40136**	**0.87339**	**1.24240**	**1.68935**
**rToiletBO_12**	**A**	**I worried about performing my bowel program.**	**2.08319**	**0.40209**	**0.91889**	**1.41131**	**1.96636**
rToiletBO_22	A	I was embarrassed by the odor associated with my bowel program	2.68325	0.48671	0.96759	1.54300	1.85690
rToiletBO_24	A	I was bothered by abdominal pain	0.92116	0.95265	1.72548	2.68904	3.62275
**rToiletBO_27**	**A**	**I worried I would have a bowel accident**	**3.16184**	**−0.00796**	**0.48797**	**1.04774**	**1.44477**
**rToiletBO_29**	**A**	**I was upset because of problems with my bowel functioning**	**2.66956**	**0.18669**	**0.67345**	**1.24687**	**1.65658**
**rToiletBO_33**	**A**	**I was frustrated by repeated bowel accidents**	**4.90259**	**0.59783**	**0.90898**	**1.35621**	**1.67546**
rToiletBO_35	A	I was bothered by fluid or loose feces leakage	3.66980	0.69274	1.04905	1.53395	1.82327
rToiletBO_36	A	I was bothered by solid stool leakage	4.15097	0.83184	1.11454	1.59355	1.88010
rToiletBO_38	B	I experienced a major inconvenience due to a bowel accident	4.29925	0.47283	0.89257	1.47547	1.72466
rToiletBO_41	B	I worried that I would have gas at an inappropriate time	1.30189	0.21713	0.85471	1.97587	2.55930
rToiletBO_44	B	My bowel care interfered with my sleep	1.86762	0.62713	1.13647	1.87682	2.36049
**rToiletBO_46**	**B**	Bowel accidents have interrupted my daily activities	**4.76412**	**0.41791**	**0.84927**	**1.43659**	**1.77930**
rToiletBO_48	B	I wanted more information on option for bowel management	1.39545	0.50884	0.84292	1.62293	2.06327
**rToiletBO_52**	**B**	I had bowel accidents	**4.01342**	**0.24540**	**0.79246**	**1.46460**	**1.87867**
rToiletBO_56	B	I was embarrassed that I needed a bowel program	1.56788	0.50277	0.92051	1.54907	1.79881
rToiletBO_60	B	I had to stop what I was doing because of a bowel accident	4.14570	0.33154	0.85888	1.52127	2.00432
rToiletBO_61	B	I spent a lot of time taking care of a bowel accident	4.63401	0.38974	0.89181	1.45725	1.92979
rToiletBO_67	B	My bowel program took 3 to 6 hours	0.99238	1.55069	2.06008	2.85185	3.68184
rToiletBO_78	B	My clothing was soiled due to a bowel accident	2.89778	0.43474	1.05601	1.92982	2.33384
rToiletBO_Com1	B	I avoided going out in public because of my bowel program	2.56380	0.73768	1.13590	1.89055	2.38333
rToiletBO_Com26.5	A	My sex life was limited by bowel issues	1.96057	1.11389	1.61193	2.08435	2.47192
**rToiletBO_Com25**	**A**	**I worried that my social activities would be interrupted by a bowel accident**	**3.21959**	**0.25994**	**0.72228**	**1.40504**	**1.78818**
rToiletBO_Com22	A	I worried that a bowel accident would disrupt my ability to work	2.53371	0.54510	0.93468	1.51278	1.97153

*Context for all items was: ‘Lately’. All items were scored 1–5. Response set A was: Not at all/A little bit/Somewhat/Quite a bit/Very much. Response set B was: Never/Rarely/Sometimes/Often/Always.

**Bold Font** indicates the items selected for the short form.

All SCI-QOL Items Copyright © 2015 David Tulsky and Kessler Foundation. All Rights Reserved. Scales should be accessed and used through the corresponding author or http://www.assessmentcenter.net. Do not modify items without permission from the copyright holder.

**Figure 2:**
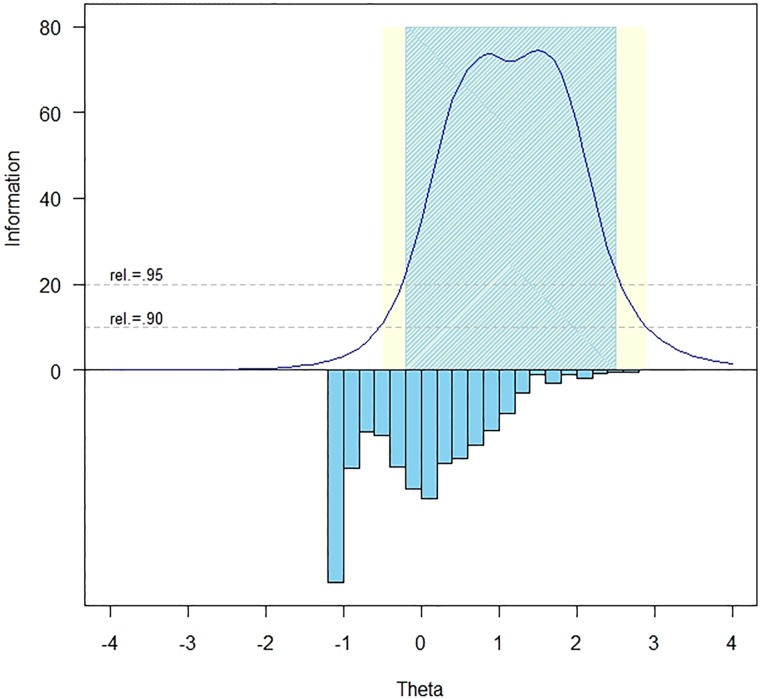
Bowel Management Difficulties item bank information and precision.

The measurement precision in the theta range between –0.3 and 2.5 is roughly equivalent to a classical reliability of 0.95 or better (Fig. 2).

All items had adequate or better S-X^2^ model fit statistics (P > 0.05), with marginal reliability equal to 0.89.

#### Differential item functioning

DIF is an indication of item bias against one subgroup. In our analyses seven items were flagged for DIF in at least one category based on the χ^2^ test; however, when the effect size measures were examined, the DIF was negligible and all 26 items were retained in the final, calibrated item bank.

### Data analysis—bladder complications

#### Preliminary analysis and item removal

As described above, 8 items related to secondary bladder complications (i.e. UTIs and kidney stones) had been removed from the Bladder Management Difficulties and these 8 items were analyzed as an individual bank using CFA and IRT methods. Prior to analyzing the 8 items, the research team removed two items that exhibited sparse responses in some categories and had low item-total correlations, signifying that they were unrelated to other items in the construct. Additionally, because 61% of the sample (*n* = 460) reported no UTIs as a complication, the research team decided against including the entire sample in the remaining analyses. Only 297 people responded more frequently than ‘Never’ to item rToiletBL_50, *‘I had a urinary tract infection’* and it was this subsample of participants that was retained for the analyses on the bladder complication items. Demographic characteristics of this subsample of 297 people may be found in Table 1. The item *‘I had a urinary tract infection’* was thus used as a screening item and was not included in the calibration analyses that included the 5 remaining items and the following results are based on this final 5-item set. Internal consistency was α = 0.72 and item/total correlations ranged from 0.38 to 0.60. In terms of test-retest reliability, Pearson's *r* was 0.70 (*n* = 245; P < 0.01 and the ICC (2,1) was 0.69 (*n* = 245; 95% CI = 0.61 to 0.76). All of the items had more than 40% of the sample selecting category of ‘1’ (Never/Not at all). No items had sparse data and there were no category inversions. No additional items were removed.

#### Dimensionality

The 5-item set demonstrated excellent fit to a unidimensional model (CFI = 0.955; RMSEA = 0.050). *R*^2^ values for 2 of the items were greater than 0.40 and 3 items were less than 0.40 (i.e. rToiletBL_28 = 0.355, rToiletBL_74 = 0.237, and rToiletBL_Com3 = 0.399). No items demonstrated LID. Eigenvalue ratio (first to second) was 3.8.

#### IRT parameter estimation and model fit

Given the reduced sample size, the 5 Bladder Complications items were calibrated using a constrained graded response model with a single (fixed) slope parameter. The slope for all items was 1.51; thresholds ranged from –0.33 to 3.33 (see Table 4).

**Table 4:** Bladder complications (scale) items and IRT parameters

Item ID	Response set*	Item stem	Item response theory calibration statistics				
			Slope	Threshold 1	Threshold 2	Threshold 3	Threshold 4
rToiletBL_21	A	A UTI (urinary tract infection) limited my daily activities	1.50995	−0.33428	0.28333	1.25659	2.03664
rToiletBL_28	A	I had an increase in spasms because of a UTI (urinary tract infection)	1.50995	−0.09740	0.43821	1.21022	1.84015
rToiletBL_74	B	I had a urinary tract infection (UTI) that would not go away	1.50995	0.23983	0.97059	1.71530	2.46737
rToiletBL_Com3	B	Bladder issues limited my sex life	1.50995	0.76965	1.23460	1.75779	2.17097
rToiletBL_Com9	B	I avoided going out because of my urinary tract infection (UTI)	1.50995	0.74972	1.46888	2.26813	3.33743

*Context for all items was: ‘Lately’. All items were scored 1–5. Response set A was: Not at all/A little bit/Somewhat/Quite a bit/Very much. Response set B was: Never/Rarely/Sometimes/Often/Always.

The Bladder Complications scale also contains a screener item, rToiletBL_50: ‘I had a urinary tract infection’ (Response set B).

All SCI-QOL Items Copyright © 2015 David Tulsky and Kessler Foundation. All Rights Reserved. Scales should be accessed and used through the corresponding author or http://www.assessmentcenter.net. Do not modify items without permission from the copyright holder.

The measurement precision at all theta levels was less than classical reliability of 0.90. All items had adequate or better S-X^2^ model fit statistics (P > 0.05), with marginal reliability equal to 0.684.

#### Differential item functioning

With the subset of individuals (*n* = 297), DIF analysis could not be performed on the Bladder Complications items.

### Short form selection and mode of administration

#### Bladder management difficulties and bowel management difficulties

Once the SCI-QOL Bladder Management Difficulties and Bowel Management Difficulties item banks were finalized, all items and parameters were programmed into the Assessment Center^SM^ ^60^ platform (www.assessmentcenter.net) and the banks can be administered as CATs or short forms (SF). The CAT administration parameters may be modified through Assessment Center^SM^, for example to reduce the standard error of score estimates, for example to maximize reliability or to reduce test burden. Fixed-length SF versions of each bank were also developed (described below). Finally, end users also have the option of selecting individual items for administration. With psychometric assistance, additional (custom) SFs can also be developed. The options for administration are described in more detail below.

The SCI-QOL utilizes the same default CAT discontinue criteria as the Patient Reported Outcomes Measurement Information System (PROMIS); the CAT minimum number of items to administer is four and the maximum is 12 with a maximum standard error of 0.3. In the default settings, therefore, the CAT will always administer at least 4 items, then will discontinue when the standard error drops below 0.3 or after the 12th item is administered.

Alternatively, the user could change the ‘discontinue criteria’ of the CAT so that it will include a larger minimum number of items to increase scoring precision, or a smaller maximum number of items to limit respondent burden. For example, administering a minimum of 8 items would result in a lengthier test, but a more reliable score would be obtained.

#### Short forms versus CATs

In some cases, it is neither possible nor practical to administer items via CAT (e.g. when computer equipment and/or internet connection are not available). To address this need, the Bladder Management Difficulties, Bowel Management Difficulties and other SCI-QOL item banks are also available as short forms. The project investigators utilized psychometric and clinical input to develop a fixed, 8-item SF version of the Bladder Management Difficulties item bank (Bladder Management Difficulties SF 8a; component items are identified by bold text in Table 2) and a 9-item short form version of the Bowel Management Difficulties item bank (Bowel Management Difficulties SF 9a; included items are identified by bold text in Table 3). The SFs were developed by selecting the most informative items throughout the range of item ‘difficulty’ (i.e. amount of the underlying trait represented by the specific item). SF scores are directly comparable to those on the CAT or full item bank given the single underlying metric. The correlations of the CATs with various stopping rules and SFs to the full bank are provided in Table 5.

**Table 5:** Bladder Management Difficulties and Bowel Management Difficulties: accuracy of variable- and fixed-length CATs and short forms: correlations with full-bank score

Item Bank	Mode	N	No. of items administered			Max	%Min	%Max	Corr. w/ Full-bank score
			Mean	SD	Min				
**Bladder** Management difficulties	Variable-length CAT (min 4)	757	8.44	3.75	4	12	32.72	20.40	0.994
	Variable-length CAT (min 8)	757	10.06	1.98	8	12	46.83	50.40	0.994
	8-item fixed-length CAT	757	8	0	8	8	n/a	n/a	0.979
	8-item short form	757	8	0	8	8	n/a	n/a	0.963
**Bowel** Management Difficulties	Variable-length CAT (min 4)	757	8.18	3.67	4	12	34.74	43.99	0.969
	Variable-length CAT (min 8)	757	9.86	1.95	8	12	50.99	43.99	0.976
	9-item fixed-length CAT	757	9	0	9	9	n/a	n/a	0.965
	9-item short form	757	9	0	9	9	n/a	n/a	0.960

Corr. = Correlation.

SFs are available for administration directly within Assessment Center, or may be downloaded in portable document format (PDF) for administration, either by paper and pencil or by an alternate electronic data capture platform or system. End users may also develop additional, custom short forms, which could then be scored on the same underlying metric.

For each IRT-calibrated bank, we compared the reliability of the full bank, fixed-length SF (8 items for Bladder and 9 for Bowel), and variable-length CAT with the default minimum of 4 items as well as with a minimum of 8 items in an effort to determine the degree of measurement precision and error associated with each method of administration. The mean, standard deviation, range, and standard error ranges for the various administration options are presented in Table 6.

**Table 6:** Bladder Management Difficulties and Bowel Management Difficulties: breadth of coverage for variable length CAT, fixed length CAT, 8-item short form, and full item bank

Item Bank	Mode	N	T score				Standard error	
			Mean ± SD	Range	% Ceiling	% Floor	Mean ± SD	Range
**Bladder** Management difficulties	Variable-length CAT (min 4)	757	49.89 ± 9.10	37.58–83.78	0.13	17.44	0.37 ± 0.15	0.21–0.63
	Variable-length CAT (min 8)	757	49.88 ± 9.11	37.58–83.78	0.13	17.44	0.36 ± 0.16	0.17–0.63
	8-item fixed-length CAT	757	49.89 ± 8.93	38.86–80.82	0.13	4.49	0.38 ± 0.18	0.17–0.64
	8-item short form	757	49.86 ± 8.72	40.90–81.20	0.13	30.65	4.21 ± 1.87	1.90–6.40
	Full bank	757	49.83 ± 9.14	37.40–84.10	0.13	13.34	3.45 ± 1.68	1.50–6.30
**Bowel** Management difficulties	Variable-length CAT (min 4)	757	48.36 ± 8.43	37.54–73.84	0.13	2.64	0.350 ± 0.15	0.17–0.56
	Variable-length CAT (min 8)	757	48.36 ± 8.46	37.54–73.85	0.13	2.64	0.328 ± 0.17	0.13–0.56
	9-item fixed-length CAT	757	48.32 ± 8.35	38.90–74.25	0.13	28.53	0.342 ± 0.19	0.12–0.59
	9-item short form	757	48.40 ± 8.31	39.20–76.30	0.40	29.99	0.363 ± 0.18	0.15–0.60
	Full bank	757	48.27 ± 8.68	36.10–77.10	0.13	15.85	0.281 ± 0.18	0.10–0.59

**Figure 3:**
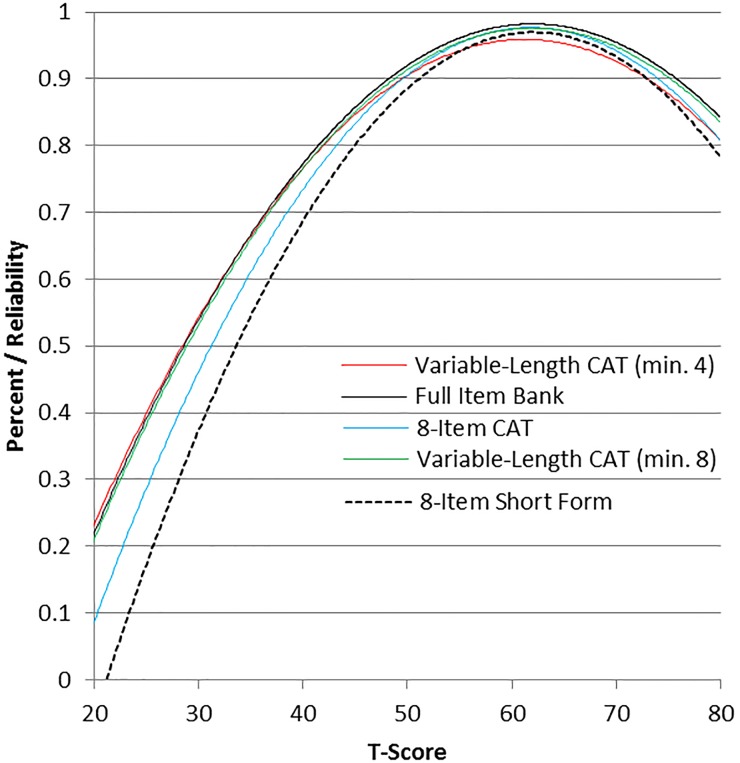
Bladder Management Difficulties: measurement reliability by T-score and assessment method.

Additionally, reliability curves for the full banks, SFs, variable length CATs (minimum of 4 items) and fixed-length CATs (8 or 9 items) may be found in Figs. 3 (Bladder) and 4 (Bowel).[Fig JSCM-D-15-00014F4]

**Figure 4 JSCM-D-15-00014F4:**
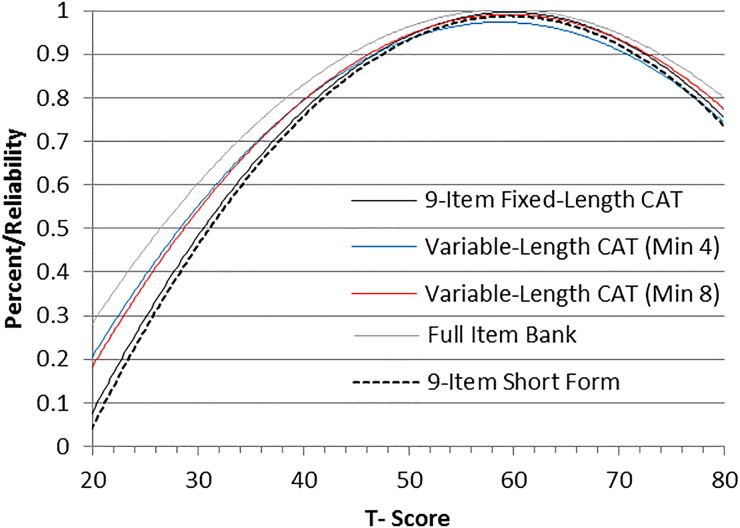
Bowel Management Difficulties: Measurement reliability by T-score and assessment method.

#### Scoring

All SCI-QOL scores were standardized on a T-metric, with a mean of 50 and a standard deviation of 10. For all Bladder and Bowel items, scoring was based on the SCI-QOL calibration data and the mean of 50 reflects the mean of an SCI population. However, a higher score does not necessarily mean better outcomes. With these CATs or SFs related to bladder and bowel difficulties, higher T scores suggest greater number of difficulties or complications. This approach was different from the other item banks that are derived from PROMIS where the score had been transformed to the PROMIS general population metric. CAT administrations of the SCI-QOL Bladder Management Difficulties and Bowel Management Difficulties item banks are scored automatically by Assessment Center^SM^. Short forms, whether administered via Assessment Center^SM^, paper and pencil, or another data capture platform, must be scored manually. An individual must complete all 8 component items in order to receive a raw score, which is a simple sum of response scores for the individual component items. Raw score to T-score conversion tables for the Bladder Management Difficulties SF8a and the Bowel Management Difficulties SF9a are provided in Tables 7 and 8, respectively.

**Table 7:** T-score conversion table for SCI-QOL Bladder Management Difficulties SF8a

Raw score	T-score	Standard error
8	40.9	6.4
9	47.6	4.3
10	49.8	4.1
11	51.8	3.7
12	53.2	3.5
13	54.8	3.0
14	56.0	2.8
15	57.0	2.6
16	58.0	2.6
17	58.9	2.5
18	59.8	2.5
19	60.6	2.4
20	61.4	2.4
21	62.2	2.4
22	62.9	2.4
23	63.7	2.4
24	64.4	2.4
25	65.2	2.4
26	65.9	2.4
27	66.7	2.4
28	67.5	2.4
29	68.3	2.5
30	69.1	2.5
31	69.9	2.6
32	70.8	2.7
33	71.8	2.8
34	72.7	2.9
35	73.8	3.1
36	74.9	3.3
37	76.1	3.4
38	77.5	3.6
39	79.1	3.9
40	81.6	4.4

**Table 8:** T-score conversion table for SCI-QOL Bowel Management Difficulties SF9a

Raw score	T-score	Standard error
9	39.2	6.0
10	45.4	3.7
11	47.3	3.5
12	49.0	3.1
13	50.2	3.0
14	51.4	2.5
15	52.4	2.4
16	53.3	2.2
17	54.0	2.1
18	54.8	2.1
19	55.5	2.0
20	56.1	2.0
21	56.8	1.9
22	57.4	1.9
23	58.0	1.9
24	58.6	1.9
25	59.2	1.9
26	59.7	1.9
27	60.3	1.9
28	60.9	1.9
29	61.4	1.9
30	62.0	1.9
31	62.6	1.9
32	63.2	1.9
33	63.7	1.9
34	64.3	1.9
35	65.0	1.9
36	65.6	1.9
37	66.3	2.0
38	67.0	2.0
39	67.7	2.1
40	68.5	2.2
41	69.4	2.4
42	70.4	2.5
43	71.6	2.7
44	73.1	3.0
45	76.3	4.0

#### Bladder complications

Due to the reduced sample size, constrained slope parameter, and lower than desired level of reliability, the investigators decided to make the Bladder Complications items available as an experimental fixed-length scale rather than as a CAT-administered item bank. Scoring of the scale (Table 9) is based on applying the IRT parameters to the calibration sample data and converting raw scores to standard scores on a T-metric. Note that participants must complete all 5 component items to receive a score. The Bladder Complications scale may be administered directly through the Assessment Center^SM^ platform or may be downloaded as a PDF file.

**Table 9:** T-score conversion table for SCI-QOL Bladder Complications scale

Raw score	T-score	Standard error
5	39.7	6.6
6	45.8	5.7
7	47.2	5.6
8	49.4	5.4
9	49.5	5.3
10	53.4	5.1
11	55.5	5.0
12	55.9	5.0
13	55.9	4.9
14	58.6	4.9
15	60.3	4.8
16	61.3	4.8
17	63.1	4.8
18	64.4	4.7
19	66.2	4.7
20	66.9	4.8
21	70.8	5.0
22	71.6	5.1
23	73.4	5.2
24	75.0	5.3
25	76.8	6.1

## Discussion

Issues related to bladder and bowel management as well as bladder complications are extremely salient to individuals with SCI. Bladder and bowel management complications can have a profound impact on one's quality of life in many ways. Our qualitative data (e.g. based upon the interviews and focus groups) highlighted the importance of this topic resulting in the development of large pools of bladder-related (*k* = 38) and bowel-related items (*k* = 52). Following our field tests, we removed several bladder items in our pool that measured secondary bladder complications and retained the items focusing solely on bladder management issues. The final SCI-QOL Bladder Management Difficulties item bank contains 15 IRT-calibrated items that could be administered as a CAT or SF. The additional items that focused on secondary bladder complications appear to have important, clinically relevant information, but only pertained to a subset of our sample (those who had experienced a UTI), which seemed to occur in only 40% of our sample. These deserve further study to identify performance issues related to sample characteristics. Because this is such an important complication after SCI, we also retained a small set of 5 items related to UTIs that can be administered as a fixed short form. Participants also spoke passionately about bowel management issues and we developed a large pool of items (*k* = 52), of which 26 items were retained in the final IRT-calibrated item bank. The SCI-QOL Bladder Management Difficulties and Bowel Management Difficulties item banks are accessible in Assessment Center^SM^ and can be administered as a CAT so that researchers and clinicians can administer only the most precise and informative items based upon an individual's responses, thus reducing patient burden. This may be clinically useful in symptom monitoring and self-management in post-acute care settings. These item banks can also be administered as a fixed length SF (conversion tables provided; Tables 6 and 7). A SCI-QOL Bladder Complications SF has been created with 5-items and is appropriate to administer if an individual responds positively to a screening items for a UTI.

To the best of our knowledge, this is the first time that a patient-centered, modern measurement theory derived approach has been used to develop a patient-reported outcome measure about bladder and bowel management (and bladder complications), specifically designed for individuals with SCI. Our formative development work using focus groups and interviews strengthened our understanding the salience of bladder and bowel management issues and their impact on all aspects of quality of life. These constructs, in the context of SCI, are extremely important to this population.

## Conclusion

To date, the assessment of patient health status in clinical practice has varied, being largely dependent on random communications between clinicians and their patients in a given episode of care. Often, there are no standardized measures being used to assess health status and complications. This lack of appropriate measures is especially problematic for patients with more severe comorbidities and functional disabilities, as in the case of SCI. The SCI-QOL builds upon the National Institute of Health (NIH) roadmap initiative and offers item banks specifically developed to assess bladder and bowel dysfunction after SCI. It provides clinicians with information about symptoms experienced by SCI patients, and provides the tools to monitor the effect of treatment and therapies to address management complications in these two key areas of care. The current article presents the development, item selection, and testing of these scales. It provides initial evidence of the reliability and validity of these items banks to be used by clinicians and researchers. This information can be used reliably to design treatment plans, and may improve communication about neurogenic bowel and bladder.

## Disclaimer statements

**Contributors** All authors have contributed significantly to the design, analysis and writing of this manuscript. The contents represent original work and have not been published elsewhere. No commercial party having a direct financial interest in the results of the research supporting this article has or will confer a benefit upon the authors or upon any organization with which the authors are associated.

**Funding** This study was supported by National Institutes of Health grant number 5R01HD054659 (Eunice Kennedy Shriver National Institute of Child Health and Human Development/National Center on Medical Rehabilitation Research and the National Institute on Neurological Disorders and Stroke).

**Conflicts of interest** No commercial party having a direct financial interest in the results of the research supporting this article has or will confer a benefit upon the authors or upon any organization with which the authors are associated.

All SCI-QOL items and parameters are Copyright © 2015 David Tulsky and the Kessler Foundation. All rights reserved. All items are freely available to the public via the Assessment Center platform (www.assessmentcenter.net). There are currently no plans for Dr. Tulsky or Kessler Foundation to profit from the use of the copyrighted material.

**Ethics approval** The Institutional Review Board at each site reviewed and approved this project.
